# Powerful Tests for Multi-Marker Association Analysis Using Ensemble Learning

**DOI:** 10.1371/journal.pone.0143489

**Published:** 2015-11-30

**Authors:** Badri Padhukasahasram, Chandan K. Reddy, Albert M. Levin, Esteban G. Burchard, L. Keoki Williams

**Affiliations:** 1 Center for Health Policy and Health Services Research, Henry Ford Health System, Detroit, Michigan, United States of America; 2 Department of Computer Science, Wayne State University, Detroit, Michigan, United States of America; 3 Department of Public Health Sciences, Henry Ford Health System, Detroit, Michigan, United States of America; 4 Department of Medicine, University of California San Francisco, San Francisco, California, United States of America; 5 Department of Bioengineering and Therapeutic Sciences, University of California San Francisco, San Francisco, California, United States of America; 6 Department of Internal Medicine, Henry Ford Health System, Detroit, Michigan, United States of America; National Taiwan University, TAIWAN

## Abstract

Multi-marker approaches have received a lot of attention recently in genome wide association studies and can enhance power to detect new associations under certain conditions. Gene-, gene-set- and pathway-based association tests are increasingly being viewed as useful supplements to the more widely used single marker association analysis which have successfully uncovered numerous disease variants. A major drawback of single-marker based methods is that they do not look at the joint effects of multiple genetic variants which individually may have weak or moderate signals. Here, we describe novel tests for multi-marker association analyses that are based on phenotype predictions obtained from machine learning algorithms. Instead of assuming a linear or logistic regression model, we propose the use of ensembles of diverse machine learning algorithms for prediction. We show that phenotype predictions obtained from ensemble learning algorithms provide a new framework for multi-marker association analysis. They can be used for constructing tests for the joint association of multiple variants, adjusting for covariates and testing for the presence of interactions. To demonstrate the power and utility of this new approach, we first apply our method to simulated SNP datasets. We show that the proposed method has the correct Type-1 error rates and can be considerably more powerful than alternative approaches in some situations. Then, we apply our method to previously studied asthma-related genes in 2 independent asthma cohorts to conduct association tests.

## Introduction

Genome wide association studies (GWAS) have generated a wealth of information about genes and genetic variants influencing various diseases and traits. [1] The vast majority of GWAS have focused on single-marker analysis and tests for significance were “corrected” for multiple hypotheses testing to obtain the correct false positive rates. Because the number of markers tested in such studies is large, a single nucleotide polymorphism (SNP) needs to have a strong effect or the sample size needs to be large enough to cross the stringent genome wide significance thresholds. Furthermore, many complex traits are thought to result from the interplay of multiple genetic and environmental factors, which are not captured by single SNP association tests. Given these limitations of single-marker analysis, many multi-marker approaches for association testing have been proposed and are increasingly being used to complement single SNP analyses [2–11].

Genes are the basic functional units of the genome and multiple polymorphisms within or near a gene can jointly affect its products. Thus, multi-marker association tests can realistically model the multiplicity that occurs biologically. While individual causal variants might show only a marginal signal of association, jointly utilizing all informative SNPs within a gene may detect their manifold effects. Testing genes also reduces the burden of multiple testing from millions of individual SNP tests to around 20,000 genes. Gene-based methods may also be less sensitive to differences in allele frequency and linkage disequilibrium patterns between population groups (and, therefore, may produce more replicable results).

To date many gene-based association tests have been proposed [4–10]. Most of these approaches first assign a subset of SNPs to a particular gene based on their location in the genome; they then seek to calculate a gene-based *p* value based on the individual SNP association tests. Versatile gene-based association study (VEGAS) is a gene-based method that combines the chi-square test statistics of individuals SNPs, while accounting for their dependence [5]. Li et al. proposed a gene-based association test that uses an extended Simes procedure (GATES). This method obtains a gene-based *p* value by integrating the *p* values of individual variants while accounting for pairwise correlations between variants when calculating the effective number of independent tests [7]. SKAT is a logistic kernel machine based test that can account for non-linear effects when determining the gene-level significance [6,8].

Generally, the methods used for combining *p* values in gene-based tests can be divided into 2 categories: best-SNP picking and all SNP aggregating tests. Best-SNP picking tests use only one SNP-based *p* value after accounting for multiple testing adjustment. GATES is an example of a testing method that falls within this category. All-SNP aggregating tests, such as VEGAS-SUM and SKAT, attempt to accumulate the effects of all SNPs into a test when determining the overall *p* value. HYST is a recently developed hybrid method that use both these kinds of approaches in its calculations [10].

Many existing gene-based approaches either use the minimum *p* value for variants within a gene or integrate the *p* values or test statistics from individual variants to determine the overall gene-level *p* values. However, this may not be optimal in terms of utilizing the information available in the data [11] and it may be better to determine the joint association of multiple predictive SNPs rather than to use individual SNP measures. In addition, many existing methods do not account for non-linear effects. Our main goal here is to develop an accurate method for multi-marker association analysis that can incorporate pairwise and higher order interactions between variables. We use phenotype prediction algorithms as a basis for constructing such association tests. Since the underlying genetic architecture of a trait and the optimal model structure for combining the association information across multiple markers are not usually known before testing, we propose a machine learning approach for this purpose. The main novelty of our approach is the use of an ensemble of diverse learning models to generate phenotype predictions. In this approach, we feed the initial predictions generated from many individual learning algorithms into a second-level learning algorithm which weights their contributions suitably to generate a final prediction [12–16]. Thus, our approach involves blending the results of different learning algorithms by using a “meta-level” learning algorithm. We also use additional variables called “meta-features” (e.g. age, gender, body mass index, individual genotypes and ancestry) as inputs to guide this blending procedure [15]. In principle, such a combination of models can allow us to better approximate (on average) the true underlying relationships between the input variables and phenotype across multiple sets of SNPs. Of note, this method allows the relationships between different groups of SNPs and the phenotype to be non-linear, complex, and variable.

We propose to use Random Forests [12–13], Support Vector machines [17–18] and linear or logistic regression as components in our ensemble learning framework. The first 2 of these methods have been widely used in numerous applications and are among the best performing prediction tools. Random forests is an ensemble learning method based on decision trees that can be used for both classification and regression. Such methods stratify the predictor space into a number of simple regions and predict using the training observations from the region to which the test observation belongs. The main idea of random forests is to build a large number of decision trees on bootstrap samples drawn from the training data. However, when building these decision trees, each time a split in a tree is considered, a random sample of *m* predictors is chosen as split candidate from the full set of *p* predictors. The split is then allowed to use only one of these *m* predictors. This has the effect of decorrelating the trees and predictions from many trees are averaged to generate the final prediction. Random forests can model non-linear relationships and interaction effects between input variables.

Support Vector Machines are supervised learning algorithms that can be used for both classification and regression. Formally, a support vector machine constructs a hyperplane in high-dimensional space based on the training data and this can be used to perform classification, and regression. Informally, the objective is to find a hyperplane that has the largest distance to training data points from any class. The larger this margin, the better is the accuracy of the classifier. In addition, SVMs have the ability to perform a non-linear classification using kernels that implicitly map their inputs into high-dimensional feature spaces.

Here, we show how machine learning algorithms can be used to construct powerful tests for multi-marker association analysis. We then show how to construct tests of association in the presence of genetic or non-genetic covariates and how to construct a multi-marker test of interactions under this framework. We first apply our method to simulated datasets to demonstrate its power and correctness. Lastly, we apply our method to previously studied asthma-related genes in two independent asthma cohorts to conduct gene-based association tests.

## Materials and Methods

### Ethics approval

The Henry Ford Health System Institutional Review Board approved this study. Patient records and information were anonymized and de-identified prior to use in this analysis.

### Approach for predicting phenotypes

Here, we present an overview of our approach to predict phenotypes from genetic and clinical variables through the use of multiple machine learning algorithms. First, we create a list of all genetic variants and clinical covariates that can potentially influence the phenotype of interest such as a disease or drug response. Next, we perform a feature selection step where we identify a subset of variables, which are useful for building a predictive model (i.e., associated with the phenotype). This can be done in many ways such as using variable importance scores from a random forest algorithm or Pearson’s correlation coefficient with the phenotype. Different machine learning algorithms (e.g., random forests [12–13], support vector machines [17–18] and logistic regression) are then trained using this subset of informative variables. Subsequently, we use the predictions from these individual models along with the selected features as inputs in a “meta-level” random forest algorithm. Lastly, we assess prediction accuracy by testing the model on an “outside the training set” and through 20-fold cross-validation.

The training and test set are created using the standard k-fold cross-validation. We randomly split a dataset with n samples into k roughly equal parts. One of these k parts becomes the test set, while the rest of the samples (k -1 parts) are used for training the algorithm and learning the model parameters. Subsequently, this model is applied to generate phenotype predictions for the samples in the test set. This process is repeated for each of the k parts to generate phenotype predictions for all the samples.

### Ensemble learning algorithm for phenotype prediction

#### Ensemble learning variation 1

1. Generate a set of all genetic variables.
2. Perform feature selection on the training data in order to identify an informative subset of variables (f_1_, f_2_…f_n_) for phenotype prediction. This can be performed using either pairwise correlation coefficients between variables and phenotype or by using random forest variable importance scores to rank the variables. Then, we can use the top 10–30% of the variables in a prediction model.
3. Train k independent machine learning approaches on the training data using the selected features and generate model predictions P_1_, P_2_…P_k_.
4. Use the predictions from step 3, P_1_, P_2_…P_k_ and f_1_, f_2_…f_n_ as inputs and train a “meta-level” learning algorithm using random forests. Note that this is a key step in the algorithm and generates a final prediction by blending many individual predictions in a possibly non-linear manner. The main goal is to learn the best model to combine individual models from the training data so that we can predict the phenotype as well as possible. The non-linear combination of models along with the meta-features gives us a more general predictive framework, which can accommodate different model structures and also allows the model to vary across the multi-dimensional parameter space.
5. Generate predictions P_blend1_ in test data using the models trained in steps 3 and then 4. Repeat for all cross-validation folds to obtain phenotype predictions for all samples.

In S1 Appendix of supporting information, we describe other variations and generalizations of this algorithm. For all calculations involving power and Type-1 error rates for our gene-based test, we use ensemble learning variation 1 with the following components: multiple linear regression or logistic regression (for quantitative or case-control traits respectively), support vector machine with linear kernel and random forests with m_try_ = 1 and n_tree_ = 1000. m_try_ is the number of SNPs to consider when creating a tree node in a random forest and n_tree_ is the number of trees used in the model. Feature selection is based on the pairwise Pearson’s correlation coefficient between variables and phenotype and we use the top 30% of the variables for constructing prediction models. The “meta-level” learning algorithm is also a random forests algorithm with m_try_ = 1 and n_tree_ = 1000 and non-linearly combines features as well as initial phenotype predictions.

The choice of m_try_ was based on testing the random forests algorithm on an empirical dataset from an asthma cohort at the Henry Ford Health System (Details can be found in [19]). We considered a large number of gene-regions from this genotype data and generated predictions for asthma drug (short-acting beta-agonist) response phenotype (a continuous variable) for varying values of m_try_. We choose the value that maximized the prediction accuracy (R^2^) on “out of the bag” samples (i.e. those not used in training) for asthma drug response.

The choice of component algorithms and parameters/settings within the ensemble learning framework is usually guided by the relative prediction accuracy (e.g. R^2^ or Mean Squared Error between observed and predicted values) for the dataset under consideration. In general, the goal is to maximize the prediction accuracy (or minimize prediction error) while maintaining a reasonable computing time and roughly correct false positive rate. We chose the parameters for each component algorithm (e.g. Random Forests, Support Vector machines) by maximizing the marginal prediction accuracy. However, there are no hard and fast rules for choosing a set of machine learning algorithms and we expect that many different variations under the ensemble learning framework (e.g. see S1 Appendix) will generate valid association tests.

### Multi-marker tests of association

Once we have estimated a model using any of the algorithms described in the previous section and predicted phenotypes for all individuals using cross-validation, we can construct tests of association in the following manner. For continuous traits, we can calculate the Pearson’s correlation coefficient between predicted (P_final_) and observed (P_actual_) values and determine the corresponding *p* values. For case-control studies, we perform a standard logistic regression that uses all genetic variables as well as P_final_ as explanatory variables and P_actual_ as response. A chi square based likelihood ratio test can then be used with respect to a null model with no variables to generate *p* values. If lik denotes the log likelihood of a model, the test statistic is given by: 2(lik_full_—lik_null_) which follows a chi square distribution with g + 1 degrees of freedom where g is the number of genetic markers.

Note that the final test statistic that we calculate, makes use of all the samples in our data and not just the testing set. The first step in our multi-marker association test is generating phenotype predictions (P_final_) for all the samples in our data. This prediction is generated using k-fold cross-validation as described previously. For case-control phenotype, the test statistic is based on a standard logistic regression model that uses all the SNPs as well as P_final_ as explanatory variables making use of all the samples. For continuous phenotype, we perform a test of correlation between P_final_ the predicted phenotype value and P_observed_ the actual phenotype values using all the samples.

### Alternate methods used for comparison

Let *p*_*(j)*_ denote the *p* value for the *j* ^th^ SNP and assume that values are in ascending order. **Let** *m* denotes the total number of SNPs in the gene. We perform simulation studies to compare the Type-1 error rate and statistical power of the ensemble learning approach with the following alternative gene-based tests:

#### Logistic regression

Each SNP is coded as 0, 1, or 2 for the number of copies of the minor allele in the genotype and the response variable is the disease status coded as 1 (case) or 0 (control). The gene-based *p* value is calculated using a chi square test statistic that is based on the log likelihood ratio comparing the full model with all available SNPs and null model with none.

#### Fisher combination test

The test statistic is given by *T = −2∑lnp*_*(j)*_, where the summation is from 1 to *m*. This statistic has a chi-square distribution with 2*m* degrees of freedom under the null hypothesis. *m* denotes the total number of SNPs in the gene and the tests are assumed to be independent. [20]

#### Original Simes test

In this test, gene-based *p* value is given by *P*_*S*_ *= min(mp*_*(j)*_ */ j)*. For independent tests, *P*_*S*_ is uniformly distributed between 0 and 1 under the null hypothesis [21]. When markers are positively correlated, the test is expected to be conservative.

#### GATES

GATES is a rapid gene-based association test that uses extended Simes procedure [7]. The test is an improvement over the original Simes procedure to account for dependency between the different SNP variables within the gene. For *m* SNPs in a gene, the overall gene-based *p* value is given by: *P*_*G*_ *= min(m*_*e*_ *p*_*(j)*_ */ m*_*e(j)*_), where *m*_*e*_ is the effective number of independent *p* values among the *m* SNPs and *m*_e(j)_ is the effective number of independent *p* values among the top *j* SNPs. *j* can vary between 1 and *m*. The effective number of tests is calculated using a formula that uses the correlation coefficients between SNP-based association test results.

#### VEGAS

A versatile gene-based test for genome-wide association studies (VEGAS) was recently proposed in [5]. The test combines SNP-based chi-square test statistics within a gene to give a gene-based test statistic. An empirical null distribution can be generated through simulation of multivariate standard normal random vectors with correlations equal to those between the SNPs in the gene. 2 versions of the VEGAS test were used here, one based on the sum of all the SNP-based chi-square statistics in the gene (VEGAS-Sum) and one based on just the largest statistic (VEGAS-Max).

#### SKAT

This test [6, 8] uses a logistic kernel machine based framework to evaluate the joint effects of multiple SNPs on a phenotype to generate a gene-based *p* value. The test also allows adjustment for covariates. The kernel machine has the ability to model complex and non-linear relationships between dependent and independent variables.

### Testing multi-marker associations in the presence of covariates

Association testing in the presence of covariates (e.g., age, gender, BMI and smoking status) can be done in the following manner. First, consider both non-genetic covariates and genetic variables together for phenotype prediction according to any of the ensemble learning algorithms described earlier. Let P_final-all_ be the predicted phenotype values. Then, remove the SNP variables and rerun the phenotype prediction algorithm. Let P_final-covariates_ be the predicted phenotype values. For continuous traits, we first calculate the Pearson’s correlation coefficient for these predicted variables with the true phenotypes (P_actual_). The strength of association for the genetic variables with continuous traits can then be calculated using the Steiger’s Z test [22] for the difference between the 2 calculated correlation coefficients. Let r_12_ and r_13_ denote the Pearson’s correlations between the true phenotype (P_actual_) and P_final-covariates_ and P_final-all_ respectively. Let r_23_ denote the Pearson’s correlation between P_final-covariates_ and P_final-all_. The Steiger’s test computes *p* values based on the following test statistic that is assumed to be standard normally distributed:

$$\text{Z}=\left({\text{Z}}_{12}-{\text{ Z}}_{13}\right)\sqrt{\text{N}-3}/\sqrt{2\text{h}\left(1-{\text{r}}_{23}\right)}$$

Here, Z_12_ and Z_13_ are Fisher’s transformations of r_12_ and r_13_, and $\text{h }=\;\left(1-{\text{ fr}}_{\text{m}}^{2}\right)\;/\;\left(1-{\text{ r}}_{\text{m}}^{2}\right)$ where $\text{f}\;=\;(1-{\text{r}}_{23})/(2-2{\text{r}}_{\text{m}}^{2})$ and ${\text{r}}_{\text{m}}^{2}\;=\;({\text{r}}_{12}^{2}+{\text{r}}_{13}^{2})/2$

For case-control studies we can use both non-genetic covariates, genetic variables, P_final-all_ and P_final-covariates_ as explanatory variables in a logistic regression model. We then use a chi square likelihood ratio test to compare the former model with a model without any genetic variables (i.e. non-genetic covariates and P_final-covariates_ only) to calculate a *p* value for the genetic contribution. If lik denotes the log likelihood of a model, the chi square test statistic is 2(lik_all_—lik_covariates_) for case-control data, which follows a chi square distribution with g + 1 degrees of freedom where g is the number of genetic markers.

### Multi-marker tests for interactions

We can test for interactions between a set of markers in the following manner. First, consider all of the SNPs together in a linear or logistic regression model (for continuous or case-control phenotype respectively) and generate phenotype predictions using cross-validation for all individuals. Let P_linear_ be the predicted phenotype values. Then, generate phenotype predictions for all individuals using any of the ensemble learning algorithms described previously. Let P_ensemble_ denote the predicted phenotype values. For continuous traits, we will use all markers as well as P_ensemble_ and P_linear_ as explanatory variables in a multiple regression model (Model 1) and perform a F test with a model (Model 0) without interactions (i.e. one with all markers and P_linear_ only) to calculate the *p* value. We compare the sum of the squared errors (SSE) of prediction to construct an F statistic with (1, N−V_Model1_−1) degrees of freedom. Here:

$$\text{F }=\;\left[{\text{SSE}}_{\text{Model}0}-{\text{ SSE}}_{\text{Model}1}\right]\left[\text{N}-{\text{ V}}_{\text{Model}1}-1\right]/{\text{SSE}}_{\text{Model}1}$$

N denotes the number of samples and V_Model1_ denotes the total number of explanatory variables in model 1. For case-control studies, we will use all markers as well as P_ensemble_ and P_linear_ as explanatory variables in a logistic regression model and perform a chi square likelihood ratio test with a model without interactions (i.e. one with all markers and P_linear_ only) to calculate the *p* value. The test statistic has 1 degree of freedom. Note that this test assumes normality of residuals and homoscedasticity. Violation of any of these assumptions may affect the Type-1 error rates for the test.

### Power and Type-1 error rates of gene-based association tests for data simulated under multiplicative and additive models

We tested the performance of the proposed gene-based test by simulating genotype data for 30 biallelic SNPs assuming Hardy Weinberg equilibrium. We assumed the following 3 scenarios of linkage disequilibrium (LD) for the 30 SNPs: i) SNPs are within blocks with high LD (r = 0.9 or 0.8 within blocks); ii) SNPs are within blocks in moderate LD (r = 0.5 or 0.4); and iii) SNPs are completely independent of one another and in linkage equilibrium. The choice of simulation settings were similar to what has been used previously [7] (Also see S1 and S2 Tables in supporting information). For each LD scenario, we considered 3 different gene sizes with the first 3, first 10 and all 30 SNPs with 1, 2 and 6 causative SNPs respectively. For each gene size, we tested the following models: i) a null model with no disease loci ii) an additive model where one SNP in each LD block had a minor allele that increased the risk additively by 0.14; and iii) a multiplicative model where one SNP in each LD block had a minor allele that increased the risk by a factor of 1.14. Disease prevalence was assumed to be 0.1. For each scenario, we used a sample of 1,500 cases and 1,500 controls drawn from a simulated population of 100,000 individuals. Type-1 error rates and statistical power for our method were obtained from 5,000 and 500 simulated case-control datasets, respectively and were based on the fraction of datasets for which the gene-based association test generated significant *p* values (i.e. *p* < 0.05).

### Power and Type-1 error rates of a gene-based association test for models with epistasis

The simulations in the previous section assumed that the effect of various disease susceptibility SNPs were independent of one another and that they increased the risk additively or multiplicatively. To explore the effect of pairwise and higher order interactions between genetic variants, we also compared the performance of methods for data simulated under models with interactions. We simulated a quantitative trait for many different models with one or more interactions among variants in addition to main effects. In addition, we also considered scenarios where there is pure epistasis (i.e. where the effect of a group of SNPs is simply due to their interactions and there are no main effects). We simulated samples of 3,000 individuals and genes with 5 or 10 SNPs assuming linkage equilibrium (See S3 Table for SNP details). The phenotype was drawn from a complex distribution involving the sum of a standard normal random variable and some multivariable function involving many SNP variables. SNP variables are coded as 0, 1 or 2. Power and Type-1 error rates were estimated based on 500 and 5,000 simulated datasets, respectively. We calculated the fraction of simulated datasets for which the gene-based method generated a significant *p* value (*p* < 0.05). We compared our result with a gene-based test using multiple linear regression, a gene-based test using GATES [7] and a gene-based test using SKAT [6, 8]. For the gene-based test with multiple linear regression, *p* values were obtained using a F test statistic.

### Power and Type-1 Error rates for a multi-marker test for interactions

For all the models simulated in the previous section, we also constructed a multi-marker test for interactions as described previously and estimated the power of such a test. We simulated samples of 3,000 individuals and genes with 5 or 10 SNPs assuming linkage equilibrium. The phenotype was drawn from a complex distribution involving the sum of a standard normal variable and interaction terms involving many SNPs. Power and Type-1 error rates were estimated based on 1,000 simulated datasets. For each model with interactions, we calculated the fraction of simulated datasets for which the multi-marker test of interactions generated a significant *p* value (*p* < 0.05); *p* values were based on an F test statistic with two parameters as described previously.

### Datasets

We applied the methods developed in this paper to data from 2 independent studies (S1 and S2 Archives in supporting information). The studies included the Study for Asthma Phenotypes and Pharmacogenomic Interactions by Race-ethnicity (SAPPHIRE) and the Genes-environments and Admixture in Latino Americans (GALA II). Recruitment for both studies is ongoing.

SAPPHIRE is population-based study which seeks to understand the genetic underpinnings of both asthma and asthma medication response. Study individuals included in this analysis were recruited from a single large health system serving the southeast Michigan and the Detroit metropolitan area. Enlisted patients with asthma met the following criteria: age 12–56 years, a physician diagnosis of asthma, and no recorded diagnosis of chronic obstructive pulmonary disease or congestive heart failure. Control individuals without asthma were recruited from a similar geographic region and were 12–56 years of age, but they did not have a prior recorded diagnosis of asthma, chronic obstructive pulmonary disease, or congestive heart failure. Genome wide genotyping was performed using the Axiom Genome-Wide AFR array (Affymetrix, Santa Clara, CA). After data quality control, genotype information was available on 586,952 SNPs for 1,099 individuals with asthma and 328 healthy controls [19]. All of the individuals from the SAPPHIRE cohort included in this analysis were African American by self-report.

The GALAII study is a case control study to identify gene-environment interactions contributing to asthma. Children of Latino descent age 8–21 years were recruited from New York City, Chicago, San Francisco, Houston, and Puerto Rico. Children with asthma had a physician diagnosis of asthma and either a 12% increase in forced expiratory volume at one second following the administration of albuterol or a positive methacholine challenge test. Genome wide genotype data was available on 3,772 individuals (1,891 with asthma and 1,881 without). Genomic DNA was genotyped on the Axiom Genome-Wide LAT array. After data cleaning, information was available for 747,075 SNPs genome wide.

### Assumptions

The following are the assumptions made in simulations for our multi-marker association test:

1. We assume that the samples are unrelated and that there is no population stratification.
2. For simulations in Tables 1–3, we assume that there are no interactions between SNPs, the genetic model is additive/multiplicative and the number of causal SNPs is small (< = 6).
3. For simulations in Tables 1–3, we assume differing levels of linkage disequilibrium between SNPs.
4. For models with epistasis (Table 4), we include both additive and recessive effects for SNPs.
5. For models with epistasis, the markers are assumed to be in linkage equilibrium.
6. For Type-1 error rates, we assume that phenotype is normally distributed for continuous traits.

**Table 1:** Comparison of empirical power and Type-1 error rates of gene-based association tests for simulated datasets assuming linkage equilibrium.

	#SNP (#DSL)	Logistic Regression	Fisher	Vegas-Sum	Original Simes	Vegas-Max	GATES	SKAT	Machine-Learning Ensemble
Linkage Equilibrium									
Type-1 Error	3(0)	4.66 [3.4–6.0]	4.67 [3.4–6.0]	4.70 [3.4–6.2]	4.61 [3.5–6.1]	4.62 [3.5–6.1]	4.61 [3.5–6.1]	5.15 [4.7–5.6]	5.94 [5.3–6.6]
Type-1 Error	10(0)	5.10 [3.8–6.7]	5.00 [3.7–6.5]	5.04 [3.8–6.5]	5.06 [3.8–6.5]	5.07 [3.8–6.5]	5.06 [3.8–6.5]	4.82 [4.4–5.3]	6.29 [5.6–7.0]
Type-1 Error	30(0)	5.26 [4.0–6.8]	4.96 [3.7–6.4]	4.97 [3.7–6.4]	4.97 [3.7–6.4]	5.04 [3.8–6.5]	4.97 [3.7–6.4]	4.86 [4.5–5.3]	4.22 [3.7–4.8]
Power Additive	3(1)	43.71 [40.7–46.8]	41.79 [38.7–44.8]	42.67 [39.6–45.7]	45.28 [42.2–48.3]	45.22 [42.2–48.3]	45.28 [42.2–48.3]	45.1 [42–48.2]	56.00 [51.5–60.4]
Power Additive	10(2)	56.88 [53.8–59.9]	53.32 [50.3–56.4]	54.56 [51.5–57.6]	54.76 [51.7–57.8]	54.00 [50.9–57.1]	54.76 [51.7–57.8]	60.8 [57.7–63.8]	57.60 [53.1–62]
Power Additive	30(6)	65.32 [62.4–68.2]	61.5 [58.4–64.5]	63.28 [60.2–66.2]	47.18 [44.1–50.3]	45.62 [42.6–48.8]	47.18 [44.1–50.3]	69.8 [66.8–72.6]	69.00 [64.7–73.0]
Power Multiplicative	3(1)	46.61 [43.5–49.8]	44.72 [41.6–47.8]	45.54 [42.5–48.7]	48.39 [45.3–51.5]	48.3 [45.2–51.5]	48.39 [45.3–51.5]	43.3 [40.2–46.4]	53.00 [48.5–57.5]
Power Multiplicative	10(2)	69.00 [66.0–71.9]	65.25 [62.3–68.2]	66.88 [63.9–69.7]	67.00 [64.0–69.9]	66.26 [63.3–69.1]	67.00 [64.0–69.9]	70.9 [68–73.7]	69.00 [64.7–73.0]
Power Multiplicative	30(6)	93.45 [91.8–94.9]	91.44 [89.6–93.1]	92.28 [90.5–93.8]	82.21 [79.8–84.5]	80.18 [77.6–82.5]	82.21 [79.8–84.5]	94.3 [92.7–95.7]	94.60 [92.2–96.4]

DSL denotes the number of disease susceptibility markers. Machine learning test is based on ensemble learning variation 1 with the following components: logistic regression, support vector machine with linear kernel and random forests with m_try_ = 1 and n_tree_ = 1000.

**Table 2:** Comparison of empirical power and Type-1 error rates of gene-based association tests in simulated datasets for moderate linkage disequilibrium.

	#SNP (#DSL)	Logistic Regression	Fisher	Vegas-Sum	Original-Simes	Vegas-Max	GATES	SKAT	Machine-Learning Ensemble
Linkage Disequilibrium									
Type-1 Error	3(0)	4.86 [3.7–6.3]	7.17 [5.7–8.9]	4.91 [3.7–6.4]	4.54 [3.4–6.0]	4.81 [3.7–6.3]	4.98 [3.7–6.4]	4.71 [4.3–5.1]	6.02 [5.4–6.7]
Type-1 Error	10(0)	4.88 [3.7–6.3]	9.8 [8.0–11.8]	4.83 [3.7–6.3]	4.55 [3.4–6.0]	4.92 [3.7–6.4]	5.00 [3.7–6.5]	4.70 [4.3–5.1]	6.16 [5.5–6.9]
Type-1 Error	30(0)	5.63 [4.4–7.2]	11.09 [9.2–13.1]	5.03 [3.8–6.5]	4.97 [3.7–6.4]	5.29 [4.0–6.8]	5.56 [4.3–7.1]	5.05 [4.6–5.5]	3.80 [3.3–4.4]
Power Additive	3(1)	44.59 [41.5–47.6]	---	49.36 [46.3–52.5]	49.71 [46.7–52.9]	50.51 [47.5–53.6]	51.23 [48.2–54.3]	46.9 [43.8–50.1]	55.20 [50.7–59.6]
Power Additive	10(2)	56.25 [53.2–59.3]	---	61.36 [58.3–64.3]	58.39 [55.3–61.4]	59.12 [56.1–62.2]	60.72 [57.7–63.7]	64.2 [61.1–67.2]	63.80 [59.4–68.0]
Power Additive	30(6)	65.47 [62.5–68.4]	---	71.96 [69.1–74.7]	53.29 [50.2–56.3]	52.24 [49.2–55.3]	55.65 [52.6–58.7]	74.3 [71.5–77]	68.00 [63.7–72.1]
Power Multiplicative	3(1)	46.52 [43.5–49.7]	---	50.98 [47.9–54.0]	51.19 [48.1–54.2]	52.00 [48.9–55.1]	52.65 [49.6–55.7]	48.0 [44.9–51.2]	53.40 [48.9–57.8]
Power Multiplicative	10(2)	68.42 [65.5–71.3]	---	72.48 [69.6–75.2]	70.66 [67.8–73.4]	70.9 [68.0–73.7]	72.4 [69.5–75.2]	75.8 [73.0–78.4]	70.20 [66–74.2]
Power Multiplicative	30(6)	93.68 [92.0–95.0]	---	95.59 [94.1–96.7]	86.07 [83.8–88.1]	84.34 [82.0–86.5]	87.52 [85.4–89.5]	94.7 [93.1–96.0]	94.70 [92.5–96.4]

DSL denotes the number of disease susceptibility markers. Machine learning test is based on ensemble learning variation 1 with the following components: logistic regression, support vector machine with linear kernel and random forests with m_try_ = 1 and n_tree_ = 1000.

**Table 3:** Comparison of empirical power and Type-1 error rates of gene-based association tests on simulated datasets for strong linkage disequilibrium.

	#SNP (#DSL)	Logistic Regression	Fisher	Vegas-Sum	Original-Simes	Vegas-Max	GATES	SKAT	Machine learning ensemble
Linkage Disequilibrium									
Type-1 Error	3(0)	4.96 [3.73–6.43]	11.49 [9.6–13.5]	5.23 [3.99–6.76]	3.88 [2.8–5.2]	5.22 [4.0–6.8]	5.35 [4.1–6.9]	4.86 [4.5–5.3]	6.05 [5.4–6.7]
Type-1 Error	10(0)	5.33 [4.08–6.88]	15.68 [13.5–18.0]	4.84 [3.7–6.3]	3.37 [2.4–4.6]	4.88 [3.7–6.3]	5.34 [4.1–6.9]	5.03 [4.6–5.5]	5.05 [4.5–5.7]
Type-1 Error	30(0)	5.57 [4.26–7.10]	17.9 [15.6–20.4]	4.89 [3.7–6.3]	3.38 [2.4–4.6]	4.89 [3.7–6.3]	5.64 [4.4–7.2]	5.04 [4.6–5.5]	3.78 [3.3–4.4]
Power Additive	3(1)	45.03 [42–48.1]	---	58.81 [55.8–61.9]	53.88 [50.8–56.9]	58.2 [55.1–61.3]	60.43 [57.4–63.5]	57.1 [54–60.2]	61.00 [56.6–65.3]
Power Additive	10(2)	57.20 [54.1–60.3]	---	75.74 [73–78.3]	66.39 [63.4–69.2]	71.71 [68.9–74.5]	74.3 [71.5–77]	77.9 [75.2–80.4]	59.00 [54.6–63.4]
Power Additive	30(6)	65.56 [62.6–68.5]	---	86.3 [84–88.4]	62.84 [59.8–65.8]	66.80 [63.8–69.7]	72.75 [69.9–75.4]	86.0 [83.7–88.1]	65.80 [61.5–70]
Power Multiplicative	3(1)	47.13 [44.1–50.3]	---	60.88 [57.8–63.8]	56.28 [53.2–59.3]	60.74 [57.7–63.7]	62.77 [59.7–65.7]	59.7 [56.6–62.8]	65.00 [60.6–69.2]
Power Multiplicative	10(2)	68.45 [65.5–71.3]	---	84.89 [82.5–87]	77.14 [74.5–79.7]	80.59 [78–82.9]	83.00 [80.5–85.3]	88.1 [85.9–90]	74.40 [70.3–78.2]
Power Multiplicative	30(6)	93.4 [91.7–94.9]	---	99.2 [98.4–99.7]	91.42 [89.6–93.1]	92.24 [90.5–93.8]	95.38 [93.9–96.5]	98.8 [97.9–99.4]	94.00 [91.5–95.9]

DSL denotes the number of disease susceptibility markers. Machine learning test is based on ensemble learning variation 1 with the following components: logistic regression, support vector machine with linear kernel and random forests with m_try_ = 1 and n_tree_ = 1000.

**Table 4:** Comparison of empirical Power and Type-1 error rates of gene-based association tests for a quantitative trait simulated under models with interactions.

Value	Phenotype distribution	#SNP (#TAS)	GATES	Linear Regression	SKAT	Machine Learning
Type-1 error	*P ~ N(0*,*1)*	5(0)	4.68 [4.11–5.30]	5.12 [4.53–5.77]	4.96 [4.37–5.60]	5.02 [4.43–5.66]
Type-1 error	*P ~ N(0*,*1)*	10(0)	5.02 [4.43–5.66]	5.02 [4.43–5.66]	4.84 [4.26–5.47]	4.22 [3.68–4.81]
Power	*P~N(0*,*1)+0*.20*snp1*snp2*snp9*snp10	10(4)	4.0 [2.46–6.11]	6.6 [4.59–9.14]	5.4 [3.59–7.76]	9.0 [6.64–11.86]
Power	*P~N(0*,*1)+0*.002*snp1 +0.002*snp2 +0.12*snp1*snp2 + 0.18*snp3*snp4	5(4)	99.6 [98.56–99.95]	99.6 [98.56–99.95]	100 [99.26–100]	97.4 [95.59–98.61]
Power	*P~N(0*,*1)+0*.25*snp1*snp2*snp3	5(3)	8.2 [5.95–10.96]	9.0 [6.64–11.86]	17.6 [14.36–21.23]	34.2 [30.05–38.54]
Power	*P ~N(0*,*1)+0*.3*snp1*snp2*snp3	5(3)	8.6 [6.29–11.41]	8.6 [6.29–11.41]	18.2 [14.91–21.87]	42.0 [37.63–46.46]
Power	*P~N(0*,*1)+0*.35*snp2*snp3*snp4	5(3)	8.8 [6.47–11.63]	9.8 [7.34–12.75]	26.4 [22.59–30.50]	55.4 [50.92–59.81]
Power	*P~N(0*,*1)+0*.65*snp1*snp2*snp3*snp8*snp9*snp10	10(6)	7.0 [4.92–9.60]	5.6 [3.75–7.99]	7.4 [5.26–10.06]	5.6 [3.75–7.99]
Power	*P~N(0*,*1)+0*.002*snp1 +0.002*snp2 + [0.2*(1+snp1)/(1+snp2)] + 0.3*snp4*snp5	5(4)	98.2 [96.61–99.17]	98.4 [96.87–99.31]	98.6 [97.14–99.44]	92.4 [89.72–94.57]
Power	*P~N(0*,*1)+0*.002*snp1 +0.002*snp2 +0.3*snp1*snp2 + 0.2*snp3*snp4	5(4)	99.8 [98.89–99.99]	100.0 [99.26–100]	100.0 [99.26–100]	100.0 [99.26–100]

TAS denotes the number of trait associated SNPs. Machine learning test is based on ensemble learning variation 1 with the following components: multiple linear regression, support vector machine with linear kernel and random forests with m_try_ = 1 and n_tree_ = 1000.

## Results

### Multiplicative and Additive models-Comparisons

Tables 1–3 shows comparisons for the performance of various methods for disease case-control datasets simulated under additive and multiplicative models. We can see that the performance of the newly proposed method based on an ensemble of machine learning algorithms is comparable to other approaches and the Type-1 error rates produced by all methods are close to what is expected. Note that when there are no disease-related SNPs in the data, we expect to see *p* values < 0.05, in around 5% of the simulated datasets due to chance alone. For the ensemble learning and logistic regression methods, we can also see that power is not strongly sensitive to the strength of linkage disequilibrium. Thus, for both additive and multiplicative models, power estimates do not appear to change much across Tables 1–3 for these methods. The running time per simulated dataset for the ensemble learning based association test was ~ 33, 43 and 49 seconds for the datasets with 3, 10 and 30 SNPs respectively on an Intel core 2.6 GHz processor machine. In S6, S7 and S8 Tables in supporting information, we show power based on the empirical values of the test statistic for the ensemble learning method.

In S4 Appendix, we have compared the distribution of the test statistic of interest for case-control data with the chi square distribution using QQ plots. These plots suggest that the chi square distribution with g+1 degrees of freedom (where g is the number of SNPs) approximates the empirical distribution of the test statistic reasonably well.

Note that for models with moderate or strong LD, the Fisher’s combination test has significantly inflated Type-1 error rate and therefore is no longer a valid test. In contrast, the VEGAS-SUM test shows the correct Type-1 error in all scenarios and appears to be significantly more powerful than the ensemble learning approach and logistic regression, when LD is strong. However, note that LD based pruning or principal component analysis can reduce the number of variables (and degrees of freedom when constructing association tests) and improve the power for both our approach (See S8 Table in supporting information) and logistic regression when markers are so strongly correlated.

### Models with epistatic effects

In Table 4, we show the power of the ensemble learning based multi-marker association test using a simulated quantitative trait for models with interactions. We compare the ensemble learning approach with a gene-based test constructed using SKAT, multiple linear regression and the extended Simes procedure (the latter implemented by GATES). Table 4 shows that our approach compares favorably with other approaches (S9 Table in supporting information shows power based on empirical value of test statistic) and that the estimated gain in power can be substantial. In Table 5, we show the power and Type-1 error rates of a multi-marker test for interactions using the same models as in Table 4. These results demonstrate the ability of the ensemble learning approach to detect the presence of interactions by testing for deviations from a linear model.

**Table 5:** Empirical Power and Type-1 error rate of a gene-based test of interactions for a simulated quantitative trait.

Value	Phenotype distribution	#SNP (#TAS)	Machine learning ensemble
Type-1 error	*P ~ N(0*,*1)*	5(0)	6.10 [4.70–7.77]
Type-1 error	*P ~ N(0*,*1)*	10(0)	5.10 [3.82–6.65]
Power	*P~N(0*,*1)+0*.20*snp1*snp2*snp9*snp10	10(4)	14.8 [12.66–17.15]
Power	*P~N(0*,*1)+0*.002*snp1 +0.002*snp2 +0.12*snp1*snp2 + 0.18*snp3*snp4	5(4)	56.3 [53.16–59.40]
Power	*P~N(0*,*1)+0*.25*snp1*snp2*snp3	5(3)	30.8 [27.95–33.76]
Power	*P ~N(0*,*1)+0*.3*snp1*snp2*snp3	5(3)	41.0 [37.93–44.12]
Power	*P~N(0*,*1)+0*.35*snp2*snp3*snp4	5(3)	59.4 [56.28–62.46]
Power	*P~N(0*,*1)+0*.65*snp1*snp2*snp3*snp8*snp9*snp10	10(6)	14.7 [12.56–17.05]
Power	*P~N(0*,*1)+0*.002*snp1 +0.002*snp2 + [0.2*(1+snp1)/(1+snp2)] + 0.3*snp4*snp5	5(4)	94.4 [92.79–95.74]
Power	*P~N(0*,*1)+0*.002*snp1 +0.002*snp2 +0.3*snp1*snp2 + 0.2*snp3*snp4	5(4)	95.8 [94.36–96.96]

TAS denotes the number of trait associated SNPs. Machine learning test is based on ensemble learning variation 1 with the following components: multiple linear regression, support vector machine with linear kernel and random forests with m_try_ = 1 and n_tree_ = 1000.

Note that the improved power of the ensemble learning approach for models with epistasis comes from its ability to model non-linearity and interactions between features. The models learned under the ensemble learning framework incorporate pairwise and possibly higher order interactions and when such effects are actually present, the machine learning based association test can be considerably more powerful than other simpler approaches which do not incorporate interactions. The predicted phenotype variable (P_final_) includes the effects of interactions and is always used when constructing the final association test and determining p values for both continuous traits and case-control data.

### Application to real datasets

We applied the proposed gene-based association test to an empirical dataset consisting of 1,427African American individuals (1,099 individuals with asthma and 328 individuals without asthma) from the SAPPHIRE cohort and 3,772 Latino children (1,891 individuals with asthma and 1,881 individuals without asthma) from the GALA II study. S4 and S5 Tables in supporting information show the sample characteristics of these populations.

We tested 9 previously studied asthma-related genes [23–25] to see if these are also associated with asthma status in our datasets. Although hundreds of genes have been implicated in asthma [25], only a few have been reliably replicated in multiple groups. Therefore, to demonstrate the performance of our method, we restricted our analysis to a small subset of asthma genes identified (and replicated in some cases) in well-powered, high-quality studies. This also reduces the burden of multiple testing. When constructing gene-based association tests, we adjusted for age, gender and the first 10 principal components in both study groups and used all available markers without performing feature selection in the ensemble learning algorithm. Principal component analysis was performed using the prcomp function in R using a random set of 10,000 markers. Tables 6 and 7 show the results of our ensemble learning gene-based association test in the SAPPHIRE and GALA II study populations, respectively. The results are compared with those obtained using the GATES method and logistic regression. At a Bonferroni adjusted significance threshold of 0.0027 (= 0.05/18 [i.e., 9 genes assessed twice]), we found that the ensemble learning gene-based test identified more statistically significant results when compared with the other gene-based methods. Specifically, *IL33* was significantly associated with asthma in Latino children using the ensemble learning gene-based test, but this gene was of borderline significance using the other 2 approaches. Analysis of local ancestry for all these tested gene-regions did not indicate any unusually high correlations with phenotypes for both the African American and Latino population samples used in this study (Results not shown here).

**Table 6:** Gene-based *p* values for previously reported asthma-related genes in 1,427 African American individuals from the SAPPHIRE cohort.

Chromosome	Gene	Length in base pairs	Number of SNPs tested	Gene-based *p* value from Ensemble Learning	Gene-based *p* value from Logistic Regression	Gene-based *p* value from GATES
1	*PYHIN1*	45513	13	0.198	0.230	0.130
2	*IL1RL1*	7466	6	0.982	0.982	0.832
5	*TSLP*	6333	5	0.063	0.064	0.533
9	*IL33*	42198	12	0.408	0.180	0.130
17	*GSDMB*	14056	15	0.401	0.401	0.870
5	*IL13*	2937	3	0.156	0.164	0.387
15	*SMAD3*	57175	23	0.323	0.323	0.359
5	*SLC22A5*	25906	15	0.0095	0.162	0.0076
5	*RAD50*	87698	34	0.010	0.010	0.367

**Table 7:** Gene-based *p* values for previously reported asthma-related genes in 3,772 Latino individuals from the GALA study.

Chromosome	Gene	Length in base pairs	Number of SNPs tested	Gene-based *p* value from Ensemble Learning	Gene-based *p* value from Logistic Regression	Gene-based *p* value from GATES
1	*PYHIN1*	45513	15	0.320	0.320	0.530
2	*IL1RL1*	7466	16	0.038	0.038	0.046
5	*TSLP*	6333	7	0.270	0.270	0.250
9	*IL33*	42198	14	0.0014	0.095	0.069
17	*GSDMB*	14056	13	2.33E-09	4.20E-08	6.24E-11
5	*IL13*	2937	10	0.280	0.280	0.100
15	*SMAD3*	57175	28	0.464	0.464	0.063
5	*SLC22A5*	25906	12	0.838	0.838	0.956
5	*RAD50*	87698	33	0.217	0.217	0.050

## Discussion

We have introduced a new method for assessing gene-based associations using genome wide genotype data. This method uses diverse machine learning algorithms to construct predictive models for the phenotype using the SNP variation within a gene and then uses these predictions to construct tests of association. Machine learning algorithms represent powerful tools for inferring the relationship between multiple explanatory variables and a phenotype while accounting for high-order interactions between the former. Because the “true” multivariable relationship between a set of variables and a trait like disease or drug response is not known in advance, we can better approximate this relationship by first learning from the data. The use of ensemble learning-based predictions leads to novel multi-marker tests of association. In addition to gene-based tests of association, these methods could also be applied for pathway-based analysis (by using phenotype predictions from individual genes as inputs) or to any other set of polymorphic variants defining a region of interest or a functional class.

There are three key advantages of using our gene-based approach compared to existing approaches. First, our method does not make *a priori* assumptions about the genetic model for a SNP (i.e. additive, recessive or dominant). When constructing our tests, we can include 3 variables for each SNP where the variants are encoded according to these 3 models (i.e. additive, recessive, dominant). Thus, we can include heterogeneous effects across SNPs. A second advantage is the ability to include any number of covariates (genetic or non-genetic) and model higher level interactions between them. This feature makes machine learning particularly suited for assessing gene-environment or gene-gene interactions. Third, creating an ensemble of diverse multivariate models with meta-features makes our method less restrictive and capable of approximating the phenotype more accurately. Collectively, these novel aspects can boost statistical power and result in novel genetic discoveries.

It should be noted that the ensemble learning based association test is not affected by the relative direction of effect of the different variants in the genes. Since the ensemble learning approach is based on generating phenotype predictions, in principle the direction of effect of particular SNPs is not expected to adversely influence the accuracy of this variable. As long as a gene contains SNPs that are associated with the phenotype, we expect that it will add to the overall prediction accuracy of the models that are learned and help us to detect associations with a gene-region. Furthermore, since the overall goal of multi-marker association tests is to find regions associated with phenotype, it is desirable to construct tests that can look at the combined effects of all variants, both common and rare [26]. Incorporating the effects of rare variants when constructing ensemble learning based multi-marker association test will be the topic of future work. One simple approach might be to combine *p* values obtained from our ensemble learning based test of common variants with any currently used rare variant multi-marker tests (e.g. SKAT) using Fisher’s method or the Original Simes procedure. Ideally, we want to include both common and rare variants (as well as structural and epigenetic variants) in an ensemble learning based phenotype prediction framework and subsequently construct association tests based on such predictions.

Extensions of these methods towards the case of multiple correlated phenotypes should also be straightforward. If instead of a single phenotype, we are interested in many phenotypes that are correlated with one another in some manner, we can construct a joint association test for all of them in the following manner. First, we will apply the ensemble learning based gene-based association test to each phenotype individually and obtain their corresponding *p* values. Subsequently, we can obtain an overall *p* value from these individual *p* values using the TATES multi-trait association method [27], which is analogous to the extended Simes procedure of GATES developed for testing multi-marker associations.

We applied our method to both simulated and empirical datasets to demonstrate its power and utility. For models without interactions between variables, the ensemble learning approach worked similarly when compared with other previous gene-based association tests. In contrast, for models dominated by interactions, our simulation studies suggested that the ensemble learning test can be considerably more powerful than other methods. Thus, for situations where epistatic or gene-environment effects are likely to be important, our association test is more likely to detect associations as compared to many of the alternative methods described.

There are a number of potential limitations to our approach that require mentioning. First, computational time can be a limitation factor when applying an ensemble learning algorithm based association test to thousands of genes. LD based pruning of SNPs, dimensionality reduction using principal component analysis within haplotype/LD blocks, and parallelization (when computing clusters are available) can all help to boost computational efficiency so that gene-based association tests can be implemented within a reasonable time. LD based pruning of SNPs or principal component analysis also ensures that the top selected features are not highly correlated with one another. When samples used are not unrelated (e.g. siblings, close relatives etc), we can estimate pairwise relatedness between individuals and subsequently choose the largest subset of people who are not too closely related to one another (e.g. no first and second degree relatives) for analysis. Next, we cannot state with certainty that the genes assessed here are involved in asthma pathogenesis, since many of these genes were identified in association studies and their function (as it relates to asthma) has not yet been elucidated. Therefore, while we assume that these genes represent true-positives, this portion of our analysis may not represent an actual demonstration of statistical power unless more detailed functional studies are conducted for the relevant genes to directly demonstrate their role in asthma. Lastly, it should also be mentioned that while our multi-marker tests can detect associations or the presence of interactive effects, they do not attempt to pinpoint the specific variants contributing to such effects.

## Conclusion

In summary, ensemble learning algorithms provide a general and flexible framework for conducting association analysis. We have shown how phenotype predictions made by such algorithms can be used for many common tasks encountered in association analysis, such as multi-marker association tests, adjusting for genetic and non-genetic covariates, and tests of interaction. Because machine learning is a highly developed area of study, prediction of response from many input variables is a well-studied problem and numerous well-established algorithms are already available which can be readily incorporated as components in an ensemble learning framework to maximize prediction accuracy and construct powerful tests of association.

## Supporting Information

S1 Appendix (DOCX)

S2 Appendix (DOCX)

S3 Appendix (DOCX)

S4 Appendix (DOCX)

S1 Archive (ZIP)

S2 Archive (ZIP)

S1 Table (DOCX)

S2 Table (DOCX)

S3 Table (DOCX)

S4 Table (DOCX)

S5 Table (DOCX)

S6 Table (DOCX)

S7 Table (DOCX)

S8 Table (DOCX)

S9 Table (DOCX)
