# Supplementary material for: Powerful Tests for Multi-Marker Association Analysis Using Ensemble Learning
Source: PLoS One. 2015 Nov 30;10(11):e0143489. doi: 10.1371/journal.pone.0143489 (PMC4664402; doi:10.1371/journal.pone.0143489)
Supplement: S1 Appendix — (DOCX) [file pone.0143489.s001.docx]

**S1 Appendix**

**Generalization: An ensemble of ensembles** Generalizations of the algorithm described previously in the methods section are also possible that can potentially further boost the prediction accuracy. In particular, an ensemble of models (steps 3 and 4 in previous algorithm) can be constructed in different ways. For example:

**Ensemble learning variation 2:** Combining of predictions from individual learning models can be done sequentially using predictions from all previous steps as inputs in the next step (i.e. instead of steps 3 and 4). Therefore, as an alternative approach, we can do the following:

i) Train learning algorithm 1 on the training data using the selected features f_1_, f_2_…f_n_ as inputs and generate model predictions P_1_.

ii) Train learning algorithm 2 on the training data using P_1_ and the selected features f_1_, f_2_…f_n_ as inputs and generate model predictions P_2_.

iii) Training learning algorithm 3 on the training data using P_1_, P_2_ and the selected features f_1_, f_2_…f_n_  as inputs and generate model predictions P_3_.

………………………………………………………………………………………………..……

…………………………………………………………………………………………………..…

k) Training learning algorithm k on the training data using P_1_, P_2_,…P_k-1_ and the selected features f_1_, f_2_…f_n_ as inputs and generate model predictions P_k_.

Note that each algorithm after the first is a meta-level learning algorithm and uses predictions from all previous algorithms as well as features (f_1_, f_2_…f_n_) as inputs to generate new predictions. Then, we generate predictions in test data P_blend2_ using the models (in the same sequence) as in training and repeat for all cross-validation folds to obtain phenotype predictions for all samples.

**Ensemble learning variation 3:** Instead of applying an ensemble learning model (variation 1) to all the samples, we can divide the high-dimensional parameter space of variables into different subsets. Then, we can train different ensemble learning models using only samples that fall in these different subsets and finally merge these models to obtain the overall prediction model. Subsequently, we can generate final predictions, P_blend3_, in test data as we did for training data for all cross-validation folds within all subsets to obtain phenotype predictions for the entire sample.

**Ensemble learning variation 4:** Other variations are also possible such as training models on residuals of previous algorithms.

Lastly, we can train a final random forest learning algorithm that uses P_1_, P_2_…P_k_, f_1_, f_2_…f_n_ and P_blend1_, P_blend2_ and P_blend3_ as inputs and performs 20-fold cross-validation to generate the final prediction P_final_.
