# Supplementary material for: Powerful Tests for Multi-Marker Association Analysis Using Ensemble Learning
Source: PLoS One. 2015 Nov 30;10(11):e0143489. doi: 10.1371/journal.pone.0143489 (PMC4664402; doi:10.1371/journal.pone.0143489)
Supplement: S2 Appendix — (DOCX) [file pone.0143489.s002.docx]

**S2 Appendix**

We also tested the performance of the ensemble learning approach under a situation where the number of cases and controls is not equal. In particular, we considered several scenarios where the number of cases is much lower than the number of controls. These results are shown in the table given below.

| Sample Size | Number of cases | Number of SNPs | Minor Allele Frequency | Number of simulations | Type 1 Error |
| --- | --- | --- | --- | --- | --- |
| 1000 | 50 | 3 | 0.3 | 10000 | 3.95 |
| 1000 | 100 | 3 | 0.3 | 10000 | 3.99 |
| 1000 | 200 | 3 | 0.3 | 10000 | 4.08 |
| 1000 | 300 | 3 | 0.3 | 10000 | 4.13 |
| 1000 | 200 | 10 | 0.3 | 10000 | 4.67 |
