# Supplementary material for: Powerful Tests for Multi-Marker Association Analysis Using Ensemble Learning
Source: PLoS One. 2015 Nov 30;10(11):e0143489. doi: 10.1371/journal.pone.0143489 (PMC4664402; doi:10.1371/journal.pone.0143489)
Supplement: S3 Appendix — (DOCX) [file pone.0143489.s003.docx]

**S3 Appendix**

Note that the simulations used in the prior tables assumed that genes have at most 30 SNPs. To check Type 1 Error rates for larger genes, we performed 2 sets of simulations in which we simulated genes with much larger number of SNPs (120 and 180 SNPs) assuming linkage equilibrium. Then, we randomly simulated phenotype values for the samples, applied the ensemble learning based multi-marker association test and then looked at the percentage of simulated datasets that yielded *p* values lower than 0.05. These results are shown below and suggest that false positive rates are not grossly inflated for our method for such larger datasets.

| Sample size | Number of Cases | Number of SNPs | Minor Allele Frequency | Number of simulations | Type 1 Error |
| --- | --- | --- | --- | --- | --- |
| 3000 | 1500 | 120 | 0.2 | 3000 | 3.80 |
| 3000 | 1500 | 180 | 0.2 | 3000 | 4.47 |
