# Supplementary material for: Powerful Tests for Multi-Marker Association Analysis Using Ensemble Learning
Source: PLoS One. 2015 Nov 30;10(11):e0143489. doi: 10.1371/journal.pone.0143489 (PMC4664402; doi:10.1371/journal.pone.0143489)

**S4 Appendix**

**QQ plots of ensemble learning test statistic and chi square distribution with relevant degrees of freedom from 5000 simulated datasets**.

1. 3 SNPs, Linkage equilibrium b) 3 SNPs, Moderate linkage disequilibrium


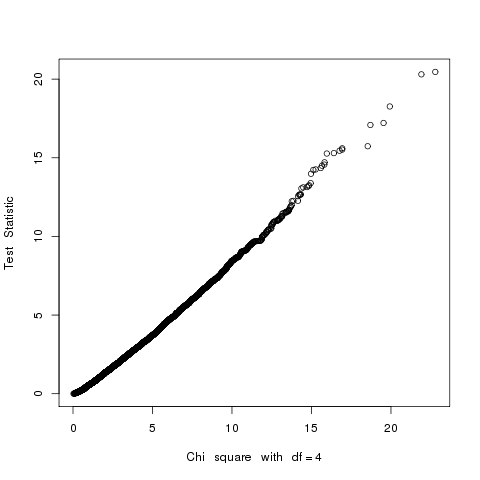

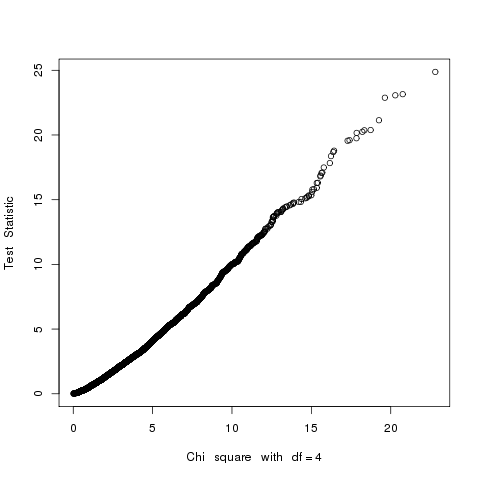


c) 10 SNPs, Linkage equilibrium d) 10 SNPs, Moderate linkage disequilibrium


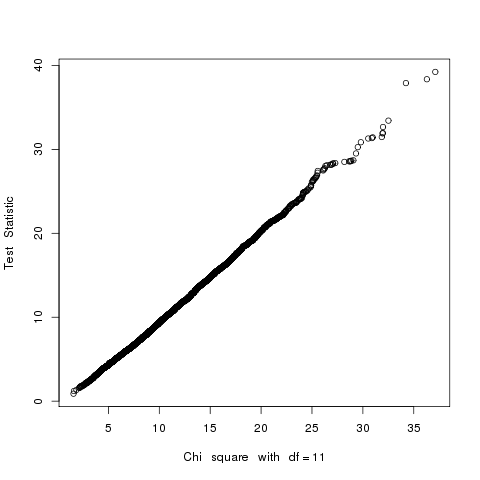

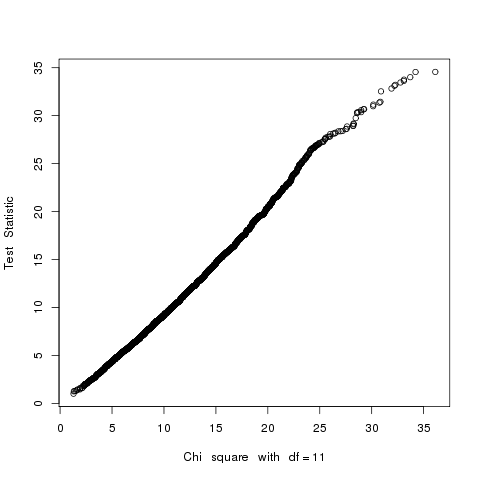


e) 30 SNPs, Linkage equilibrium f) 30 SNPs, Moderate linkage disequilibrium


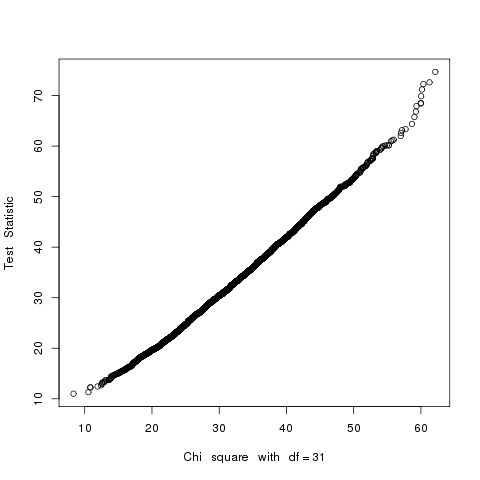

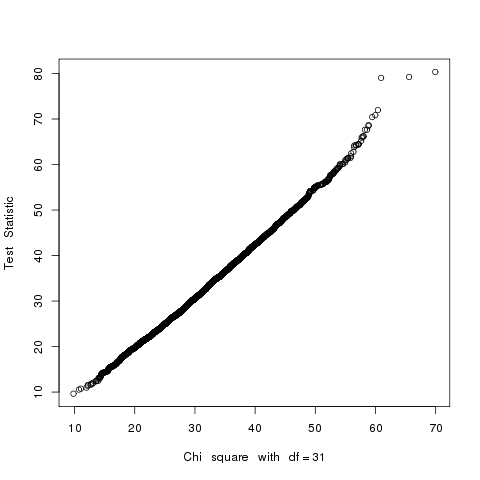

Supplement: S4 Appendix — (DOCX) [file pone.0143489.s004.docx]
