# Supplementary material for: Powerful Tests for Multi-Marker Association Analysis Using Ensemble Learning
Source: PLoS One. 2015 Nov 30;10(11):e0143489. doi: 10.1371/journal.pone.0143489 (PMC4664402; doi:10.1371/journal.pone.0143489)
Supplement: S2 Table — (DOCX) [file pone.0143489.s008.docx]

**S2 Table. Minor allele frequencies (MAF), disease susceptibility loci (DSL), pairwise correlation coefficient (r) for SNPs with moderate LD.**

| **SNP** | **DSL** | **MAF** | **r** | **r** | **r** | **r** | **r** | **r** | **r** | **r** | **r** | **r** | **r** | **r** | **r** | **r** | **r** | **r** | **r** | **r** | **r** | **r** | **r** | **r** | **r** | **r** | **r** | **r** | **r** | **r** | **r** | **r** |
| --- | --- | --- | --- | --- | --- | --- | --- | --- | --- | --- | --- | --- | --- | --- | --- | --- | --- | --- | --- | --- | --- | --- | --- | --- | --- | --- | --- | --- | --- | --- | --- | --- |
| 1 | Y | 0.20 | 1 | .5 | .5 | .5 | .5 | .5 | 0 | 0 | 0 | 0 | 0 | 0 | 0 | 0 | 0 | 0 | 0 | 0 | 0 | 0 | 0 | 0 | 0 | 0 | 0 | 0 | 0 | 0 | 0 | 0 |
| 2 |  | 0.20 |  | 1 | .5 | .5 | .5 | .5 | 0 | 0 | 0 | 0 | 0 | 0 | 0 | 0 | 0 | 0 | 0 | 0 | 0 | 0 | 0 | 0 | 0 | 0 | 0 | 0 | 0 | 0 | 0 | 0 |
| 3 |  | 0.20 |  |  | 1 | .5 | .5 | .5 | 0 | 0 | 0 | 0 | 0 | 0 | 0 | 0 | 0 | 0 | 0 | 0 | 0 | 0 | 0 | 0 | 0 | 0 | 0 | 0 | 0 | 0 | 0 | 0 |
| 4 |  | 0.20 |  |  |  | 1 | .5 | .5 | 0 | 0 | 0 | 0 | 0 | 0 | 0 | 0 | 0 | 0 | 0 | 0 | 0 | 0 | 0 | 0 | 0 | 0 | 0 | 0 | 0 | 0 | 0 | 0 |
| 5 |  | 0.20 |  |  |  |  | 1 | .5 | 0 | 0 | 0 | 0 | 0 | 0 | 0 | 0 | 0 | 0 | 0 | 0 | 0 | 0 | 0 | 0 | 0 | 0 | 0 | 0 | 0 | 0 | 0 | 0 |
| 6 |  | 0.20 |  |  |  |  |  | 1 | 0 | 0 | 0 | 0 | 0 | 0 | 0 | 0 | 0 | 0 | 0 | 0 | 0 | 0 | 0 | 0 | 0 | 0 | 0 | 0 | 0 | 0 | 0 | 0 |
| 7 |  | 0.40 |  |  |  |  |  |  | 1 | .4 | .4 | .4 | 0 | 0 | 0 | 0 | 0 | 0 | 0 | 0 | 0 | 0 | 0 | 0 | 0 | 0 | 0 | 0 | 0 | 0 | 0 | 0 |
| 8 |  | 0.40 |  |  |  |  |  |  |  | 1 | .4 | .4 | 0 | 0 | 0 | 0 | 0 | 0 | 0 | 0 | 0 | 0 | 0 | 0 | 0 | 0 | 0 | 0 | 0 | 0 | 0 | 0 |
| 9 |  | 0.40 |  |  |  |  |  |  |  |  | 1 | .4 | 0 | 0 | 0 | 0 | 0 | 0 | 0 | 0 | 0 | 0 | 0 | 0 | 0 | 0 | 0 | 0 | 0 | 0 | 0 | 0 |
| 10 | Y | 0.40 |  |  |  |  |  |  |  |  |  | 1 | 0 | 0 | 0 | 0 | 0 | 0 | 0 | 0 | 0 | 0 | 0 | 0 | 0 | 0 | 0 | 0 | 0 | 0 | 0 | 0 |
| 11 | Y | 0.25 |  |  |  |  |  |  |  |  |  |  | 1 | .5 | .5 | .5 | .5 | .5 | 0 | 0 | 0 | 0 | 0 | 0 | 0 | 0 | 0 | 0 | 0 | 0 | 0 | 0 |
| 12 |  | 0.25 |  |  |  |  |  |  |  |  |  |  |  | 1 | .5 | .5 | .5 | .5 | 0 | 0 | 0 | 0 | 0 | 0 | 0 | 0 | 0 | 0 | 0 | 0 | 0 | 0 |
| 13 |  | 0.25 |  |  |  |  |  |  |  |  |  |  |  |  | 1 | .5 | .5 | .5 | 0 | 0 | 0 | 0 | 0 | 0 | 0 | 0 | 0 | 0 | 0 | 0 | 0 | 0 |
| 14 |  | 0.25 |  |  |  |  |  |  |  |  |  |  |  |  |  | 1 | .5 | .5 | 0 | 0 | 0 | 0 | 0 | 0 | 0 | 0 | 0 | 0 | 0 | 0 | 0 | 0 |
| 15 |  | 0.25 |  |  |  |  |  |  |  |  |  |  |  |  |  |  | 1 | .5 | 0 | 0 | 0 | 0 | 0 | 0 | 0 | 0 | 0 | 0 | 0 | 0 | 0 | 0 |
| 16 |  | 0.25 |  |  |  |  |  |  |  |  |  |  |  |  |  |  |  | 1 | 0 | 0 | 0 | 0 | 0 | 0 | 0 | 0 | 0 | 0 | 0 | 0 | 0 | 0 |
| 17 |  | 0.30 |  |  |  |  |  |  |  |  |  |  |  |  |  |  |  |  | 1 | .4 | .4 | .4 | 0 | 0 | 0 | 0 | 0 | 0 | 0 | 0 | 0 | 0 |
| 18 |  | 0.30 |  |  |  |  |  |  |  |  |  |  |  |  |  |  |  |  |  | 1 | .4 | .4 | 0 | 0 | 0 | 0 | 0 | 0 | 0 | 0 | 0 | 0 |
| 19 |  | 0.30 |  |  |  |  |  |  |  |  |  |  |  |  |  |  |  |  |  |  | 1 | .4 | 0 | 0 | 0 | 0 | 0 | 0 | 0 | 0 | 0 | 0 |
| 20 | Y | 0.30 |  |  |  |  |  |  |  |  |  |  |  |  |  |  |  |  |  |  |  | 1 | 0 | 0 | 0 | 0 | 0 | 0 | 0 | 0 | 0 | 0 |
| 21 | Y | 0.10 |  |  |  |  |  |  |  |  |  |  |  |  |  |  |  |  |  |  |  |  | 1 | .5 | .5 | .5 | .5 | .5 | 0 | 0 | 0 | 0 |
| 22 |  | 0.10 |  |  |  |  |  |  |  |  |  |  |  |  |  |  |  |  |  |  |  |  |  | 1 | .5 | .5 | .5 | .5 | 0 | 0 | 0 | 0 |
| 23 |  | 0.10 |  |  |  |  |  |  |  |  |  |  |  |  |  |  |  |  |  |  |  |  |  |  | 1 | .5 | .5 | .5 | 0 | 0 | 0 | 0 |
| 24 |  | 0.10 |  |  |  |  |  |  |  |  |  |  |  |  |  |  |  |  |  |  |  |  |  |  |  | 1 | .5 | .5 | 0 | 0 | 0 | 0 |
| 25 |  | 0.10 |  |  |  |  |  |  |  |  |  |  |  |  |  |  |  |  |  |  |  |  |  |  |  |  | 1 | .5 | 0 | 0 | 0 | 0 |
| 26 |  | 0.10 |  |  |  |  |  |  |  |  |  |  |  |  |  |  |  |  |  |  |  |  |  |  |  |  |  | 1 | 0 | 0 | 0 | 0 |
| 27 |  | 0.15 |  |  |  |  |  |  |  |  |  |  |  |  |  |  |  |  |  |  |  |  |  |  |  |  |  |  | 1 | .4 | .4 | .4 |
| 28 |  | 0.15 |  |  |  |  |  |  |  |  |  |  |  |  |  |  |  |  |  |  |  |  |  |  |  |  |  |  |  | 1 | .4 | .4 |
| 29 |  | 0.15 |  |  |  |  |  |  |  |  |  |  |  |  |  |  |  |  |  |  |  |  |  |  |  |  |  |  |  |  | 1 | .4 |
| 30 | Y | 0.15 |  |  |  |  |  |  |  |  |  |  |  |  |  |  |  |  |  |  |  |  |  |  |  |  |  |  |  |  |  | 1 |
