# Supplementary material for: Powerful Tests for Multi-Marker Association Analysis Using Ensemble Learning
Source: PLoS One. 2015 Nov 30;10(11):e0143489. doi: 10.1371/journal.pone.0143489 (PMC4664402; doi:10.1371/journal.pone.0143489)
Supplement: S3 Table — (DOCX) [file pone.0143489.s009.docx]

**S3 Table. Minor allele frequencies, inheritance mode and disease susceptibility SNPs for models with epistasis. TAS denotes trait associated SNP, A denotes additive and R denotes recessive. SNPs are assumed to be in linkage equilibrium in all of these models.**

| Phenotype distribution | #SNP  (#TAS) | MAF | SNP1 | SNP2 | SNP3 | SNP4 | SNP5 | SNP6 | SNP7 | SNP8 | SNP9 | SNP10 |
| --- | --- | --- | --- | --- | --- | --- | --- | --- | --- | --- | --- | --- |
| *P ~ N(0,1)* | 5(0) | 0.3 | A | A | A | A | A | - | - | - | - | - |
| *P ~ N(0,1)* | 10(0) | 0.3 | A | A | A | A | A | A | A | A | A | A |
| *P~N(0,1)+0.20*snp1*snp2*snp9*snp10* | 10(4) | 0.3 | R | R | A | A | A | A | A | A | A | A |
| *P~N(0,1)+0.002*snp1 +0.002*snp2 +0.12*snp1*snp2 + 0.18*snp3*snp4* | 5(4) | 0.3 | R | R | A | A | A | - | - | - | - | - |
| *P~N(0,1)+0.25*snp1*snp2*snp3* | 5(3) | 0.3 | R | R | A | A | A | - | - | - | - | - |
| *P ~N(0,1)+0.3*snp1*snp2*snp3* | 5(3) | 0.3 | R | R | A | A | A | - | - | - | - | - |
| *P~N(0,1)+0.35*snp2*snp3*snp4* | 5(3) | 0.3 | R | R | A | A | A | - | - | - | - | - |
| *P~N(0,1)+0.65*snp1*snp2*snp3*snp8*snp9*snp10* | 10(6) | 0.3 | R | R | A | A | A | A | A | A | A | A |
| *P~N(0,1)+0.002*snp1 +0.002*snp2 + [0.2*(1+snp1)/(1+snp2)] + 0.3*snp4*snp5* | 5(4) | 0.1 | A | A | A | A | A | - | - | - | - | - |
| *P~N(0,1)+0.002*snp1 +0.002*snp2 +0.3*snp1*snp2 + 0.2*snp3*snp4* | 5(4) | 0.3 | R | R | A | A | A | - | - | - | - | - |
