# Supplementary material for: Powerful Tests for Multi-Marker Association Analysis Using Ensemble Learning
Source: PLoS One. 2015 Nov 30;10(11):e0143489. doi: 10.1371/journal.pone.0143489 (PMC4664402; doi:10.1371/journal.pone.0143489)
Supplement: S4 Table — (DOCX) [file pone.0143489.s010.docx]

**S4 Table. Sample characteristics of the SAPPHIRE cohort.**

| **Variable** | **Metric** | **Healthy African American individuals**  **(n=328)** | **African American individuals with asthma**  **(n=1,073)** |
| --- | --- | --- | --- |
| Age (years) | Mean + SD | 41.23 ± 13.28 | 31.65 ± 14.57 |
| Females | Counts (percentage) | 212 (64.63%) | 671 (62.53%) |
| Body mass index (kg/m^2^) | Mean + SD | 32.19 ± 7.58 | 31.49 ± 9.07 |
| Smoking status |  |  |  |
| Never | Counts (percentage) | 239 (72.8%) | 893 (83.2%) |
| Past | Counts (percentage) | 33 (10.1%) | 96 (8.9%) |
| Current | Counts (percentage) | 56 (17.1%) | 84 (7.8%) |
| Asthma age of onset (years) | Mean + SD | -- | 12.65 ± 13.55 |
| Forced Expiratory Volume(FEV_1_) | Mean + SD | 2.74 ± 0.71 | 2.58 ± 0.75 |
| Percent of predicted FEV_1_ | Mean + SD | 97.6 ± 15.3 | 87.9 ± 18.4 |
| Percent change in Short-acting beta-agonist response | Mean + SD | 2.51 ± 7.95 | 10.53 ± 12.93 |
