# Supplementary material for: Powerful Tests for Multi-Marker Association Analysis Using Ensemble Learning
Source: PLoS One. 2015 Nov 30;10(11):e0143489. doi: 10.1371/journal.pone.0143489 (PMC4664402; doi:10.1371/journal.pone.0143489)
Supplement: S5 Table — (DOCX) [file pone.0143489.s011.docx]

**S5 Table. Sample characteristics of the GALAII cohort.**

| **Variable** | **Metric** | **Healthy Latino children**  **(n = 1,881)** | **Latino children with asthma**  **(n = 1,891)** |
| --- | --- | --- | --- |
| Age (years) | Mean + SD | 13.65 + 3.50 | 12.53 + 3.25 |
| Females | Counts (percentage) | 1059 (56.29%) | 845 (44.68%) |
| Body Mass index (kg/m^2^) | Mean + SD | 24.40 + 6.87 | 23.09 + 6.51 |
| Smoking status |  |  |  |
| Smoker | Counts (percentage) | 92 (4.89%) | 57(3.01%) |
| Non-smoker | Counts (percentage) | 1789 (95.11%) | 1834(96.99%) |
| Ethnicity |  |  |  |
| Mexican | Counts (percentage) | 661 (35.14%) | 596 (31.52%) |
| Puerto Rican | Counts (percentage) | 894 (47.53%) | 892 (47.17%) |
| Spanish | Counts (percentage) | 125 (6.64%) | 244 (12.90%) |
| Other | Counts (percentage) | 201 (10.69%) | 159 (8.41%) |
