# Supplementary material for: Powerful Tests for Multi-Marker Association Analysis Using Ensemble Learning
Source: PLoS One. 2015 Nov 30;10(11):e0143489. doi: 10.1371/journal.pone.0143489 (PMC4664402; doi:10.1371/journal.pone.0143489)
Supplement: S6 Table — (DOCX) [file pone.0143489.s012.docx]

**S6 Table. Comparison of power of gene-based association tests on simulated datasets for linkage equilibrium. Power for machine learning based on empirical distribution of test statistic from 5000 simulations.**

|  | #SNP  (#DSL) | Logistic Regression | Fisher | Vegas-Sum | Original  Simes | Vegas-Max | GATES | SKAT | Ensemble learning |
| --- | --- | --- | --- | --- | --- | --- | --- | --- | --- |
| Linkage Equilibrium | | | | | | | | | |
| Power  Additive | 3(1) | 43.71  [40.7-46.8] | 41.79  [38.7-44.8] | 42.67  [39.6-45.7] | 45.28  [42.2-48.3] | 45.22  [42.2-48.3] | 45.28  [42.2-48.3] | 45.1  [42-48.2] | 42.40  [39.3-45.5] |
| Power  Additive | 10(2) | 56.88  [53.8-59.9] | 53.32  [50.3-56.4] | 54.56  [51.5-57.6] | 54.76  [51.7-57.8] | 54.00  [50.9-57.1] | 54.76  [51.7-57.8] | 60.8  [57.7-63.8] | 53.40  [50.2-56.5] |
| Power  Additive | 30(6) | 65.32  [62.4-68.2] | 61.5  [58.4-64.5] | 63.28  [60.2-66.2] | 47.18  [44.1-50.3] | 45.62  [42.6-48.8] | 47.18  [44.1-50.3] | 69.8  [66.8-72.6] | 65.20  [62.1-68.1] |
| Power  Multiplicative | 3(1) | 46.61  [43.5-49.8] | 44.72  [41.6-47.8] | 45.54  [42.5-48.7] | 48.39  [45.3-51.5] | 48.3  [45.2-51.5] | 48.39  [45.3-51.5] | 43.3  [40.2-46.4] | 46.1  [42.9-49.2] |
| Power  Multiplicative | 10(2) | 69.00  [66.0-71.9] | 65.25  [62.3-68.2] | 66.88  [63.9-69.7] | 67.00  [64.0-69.9] | 66.26  [63.3-69.1] | 67.00  [64.0-69.9] | 70.9  [68-73.7] | 67.00  [63.9-69.9] |
| Power  Multiplicative | 30(6) | 93.45  [91.8-94.9] | 91.44  [89.6-93.1] | 92.28  [90.5-93.8] | 82.21  [79.8-84.5] | 80.18  [77.6-82.5] | 82.21  [79.8-84.5] | 94.3  [92.7-95.7] | 92.70  [90.9-94.2] |

DSL denotes the number of disease susceptibility markers. Machine learning test is based on ensemble learning variation 1 with the following components: logistic regression, support vector machine with linear kernel and random forests with m_try_ = 1 and n_tree_ = 1000.
