# Supplementary material for: Powerful Tests for Multi-Marker Association Analysis Using Ensemble Learning
Source: PLoS One. 2015 Nov 30;10(11):e0143489. doi: 10.1371/journal.pone.0143489 (PMC4664402; doi:10.1371/journal.pone.0143489)
Supplement: S7 Table — (DOCX) [file pone.0143489.s013.docx]

**S7 Table. Comparison of power of gene-based association tests on simulated datasets for moderate linkage disequilibrium. Power for machine learning based on empirical distribution of test statistic from 5000 simulations.**

|  | #SNP  (#DSL) | Logistic Regression | Fisher | Vegas-Sum | Original-Simes | Vegas-Max | GATES | SKAT | Ensemble  learning |
| --- | --- | --- | --- | --- | --- | --- | --- | --- | --- |
| Moderate Linkage Disequilibrium | | | | | | | | | |
| Power  Additive | 3(1) | 44.59  [41.5-47.6] | --- | 49.36  [46.3-52.5] | 49.71  [46.7-52.9] | 50.51  [47.5-53.6] | 51.23  [48.2-54.3] | 46.9  [43.8-50.1] | 55.7  [52.5-58.8] |
| Power  Additive | 10(2) | 56.25  [53.2-59.3] | --- | 61.36  [58.3-64.3] | 58.39  [55.3-61.4] | 59.12  [56.1-62.2] | 60.72  [57.7-63.7] | 64.2  [61.1-67.2] | 62.0  [58.9-65.0] |
| Power  Additive | 30(6) | 65.47  [62.5-68.4] | --- | 71.96  [69.1-74.7] | 53.29  [50.2-56.3] | 52.24  [49.2-55.3] | 55.65  [52.6-58.7] | 74.3  [71.5-77] | 70.2  [67.2-73.0] |
| Power  Multiplicative | 3(1) | 46.52  [43.5-49.7] | --- | 50.98  [47.9-54.0] | 51.19  [48.1-54.2] | 52.00  [48.9-55.1] | 52.65  [49.6-55.7] | 48.0  [44.9-51.2] | 56.2  [53.0-59.3] |
| Power  Multiplicative | 10(2) | 68.42  [65.5-71.3] | --- | 72.48  [69.6-75.2] | 70.66  [67.8-73.4] | 70.9  [68.0-73.7] | 72.4  [69.5-75.2] | 75.8  [73.0-78.4] | 74.3  [71.4-76.9] |
| Power  Multiplicative | 30(6) | 93.68  [92.0-95.0] | --- | 95.59  [94.1-96.7] | 86.07  [83.8-88.1] | 84.34  [82.0-86.5] | 87.52  [85.4-89.5] | 94.7  [93.1-96.0] | 95.3  [93.7-96.5] |

DSL denotes the number of disease susceptibility markers. Machine learning test is based on ensemble learning variation 1 with the following components: logistic regression, support vector machine with linear kernel and random forests with m_try_ = 1 and n_tree_ = 1000.
