# Supplementary material for: Powerful Tests for Multi-Marker Association Analysis Using Ensemble Learning
Source: PLoS One. 2015 Nov 30;10(11):e0143489. doi: 10.1371/journal.pone.0143489 (PMC4664402; doi:10.1371/journal.pone.0143489)
Supplement: S8 Table — (DOCX) [file pone.0143489.s014.docx]

**S8 Table. Comparison of power of gene-based association tests on simulated datasets for strong linkage disequilibrium. Power for machine learning based on empirical distribution of test statistic from 5000 simulations.**

|  | #SNP  (#DSL) | Logistic Regression | Fisher | Vegas-Sum | Original-Simes | Vegas-Max | GATES | SKAT | Ensemble learning |
| --- | --- | --- | --- | --- | --- | --- | --- | --- | --- |
| Strong Linkage Disequilibrium | | | | | | | | | |
| Power  Additive | 3(1) | 45.03  [42-48.1] | --- | 58.81  [55.8-61.9] | 53.88  [50.8-56.9] | 58.2  [55.1-61.3] | 60.43  [57.4-63.5] | 57.1  [54-60.2] | 44.4  [41.2-47.5] |
| Power  Additive | 10(2) | 57.20  [54.1-60.3] | --- | 75.74  [73-78.3] | 66.39  [63.4-69.2] | 71.71  [68.9-74.5] | 74.3  [71.5-77] | 77.9  [75.2-80.4] | 67.4  [64.3-70.3] |
| Power  Additive | 30(6) | 65.56  [62.6-68.5] | --- | 86.3  [84-88.4] | 62.84  [59.8-65.8] | 66.80  [63.8-69.7] | 72.75  [69.9-75.4] | 86.0  [83.7-88.1] | 83.9  [81.4-86.1] |
| Power  Multiplicative | 3(1) | 47.13  [44.1-50.3] | --- | 60.88  [57.8-63.8] | 56.28  [53.2-59.3] | 60.74  [57.7-63.7] | 62.77  [59.7-65.7] | 59.7  [56.6-62.8] | 45.8  [42.6-48.9] |
| Power  Multiplicative | 10(2) | 68.45  [65.5-71.3] | --- | 84.89  [82.5-87] | 77.14  [74.5-79.7] | 80.59  [78-82.9] | 83.00  [80.5-85.3] | 88.1  [85.9-90]] | 79.6  [76.9-82.0] |
| Power  Multiplicative | 30(6) | 93.4  [91.7-94.9] | --- | 99.2  [98.4-99.7] | 91.42  [89.6-93.1] | 92.24  [90.5-93.8] | 95.38  [93.9-96.5] | 98.8  [97.9-99.4] | 99.1  [98.2-99.5] |

DSL denotes the number of disease susceptibility markers. Machine learning test is based on ensemble learning variation 1 with the following components: logistic regression, support vector machine with linear kernel and random forests with m_try_ = 1 and n_tree_ = 1000. Rather than use the original SNPs, we used only the top 2, top 3 and top 6 principal components respectively as variables for the 3 SNP, 10 SNP and 30 SNP datasets when calculating the test statistic for this scenario.
