# Supplementary material for: Powerful Tests for Multi-Marker Association Analysis Using Ensemble Learning
Source: PLoS One. 2015 Nov 30;10(11):e0143489. doi: 10.1371/journal.pone.0143489 (PMC4664402; doi:10.1371/journal.pone.0143489)
Supplement: S9 Table — (DOCX) [file pone.0143489.s015.docx]

**S9 Table. Comparison of power of gene-based association tests for models with interactions. Power for machine learning based on empirical distribution of test statistic from 5000 simulations.**

| Value | Phenotype distribution | #SNP  (#TAS) | GATES | Linear  Regression | SKAT | Ensemble learning |
| --- | --- | --- | --- | --- | --- | --- |
| Power | *P~N(0,1)+0.20*snp1*snp2*snp9*snp10* | 10(4) | 6.0  [4.0-8.4] | 6.0  [4.0-8.4] | 6.6  [4.5-9.1] | 5.8  [3.9-8.2] |
| Power | *P~N(0,1)+0.002*snp1 +0.002*snp2 +0.12*snp1*snp2 + 0.18*snp3*snp4* | 5(4) | 99.6  [98.5-99.9] | 99.6  [98.5-99.9] | 100  [99.2-100.0] | 97.4  [95.5-98.6] |
| Power | *P~N(0,1)+0.25*snp1*snp2*snp3* | 5(3) | 7.4  [5.2-10.0] | 8.8  [6.4-11.6] | 15.0  [11.9-18.4] | 25.4  [21.6-29.4] |
| Power | *P ~N(0,1)+0.3*snp1*snp2*snp3* | 5(3) | 10.8  [8.2-13.8] | 12.0  [9.2-15.1] | 18.6  [15.2-22.2] | 39.0  [34.7-43.4] |
| Power | *P~N(0,1)+0.35*snp2*snp3*snp4* | 5(3) | 8.8  [6.4-11.6] | 9.8  [7.3-12.7] | 26.4  [22.5-30.4] | 54.0  [49.5-58.4] |
| Power | *P~N(0,1)+0.45*snp2*snp3*snp4* | 5(3) | 18.0  [14.7-21.6] | 21.2  [17.6-25.0] | 43.8  [39.3-48.2] | 80.2  [76.4-83.6] |
| Power | *P~N(0,1)+0.65*snp1*snp2*snp3*snp8*snp9*snp10* | 10(6) | 5.8  [3.9-8.2] | 4.0  [2.4-6.1] | 7.8  [5.6-10.5] | 6.8  [4.7-9.3] |
| Power | *P~N(0,1)+0.002*snp1 +0.002*snp2 + [0.2*(1+snp1)/(1+snp2)] + 0.3*snp4*snp5* | 5(4) | 98.2  [96.6-99.1] | 99.6  [98.5-99.9] | 99.6  [98.5-99.9] | 92.8  [90.1-94.9] |
| Power | *P~N(0,1)+0.002*snp1 +0.002*snp2 +0.3*snp1*snp2 + 0.2*snp3*snp4* | 5(4) | 99.8  [98.8-99.9] | 100.0  [99.2-100.0] | 100.0  [99.2-100.0] | 100.0  [99.2-100.0] |

TAS denotes the number of trait associated SNPs. Machine learning test is based on ensemble learning variation 1 with the following components: multiple linear regression, support vector machine with linear kernel and random forests with m_try_ = 1 and n_tree_ = 1000.
